# Hepatitis B virus whole-X and X protein play distinct roles in HBV-related hepatocellular carcinoma progression

**DOI:** 10.1186/s13046-016-0366-3

**Published:** 2016-06-03

**Authors:** Yu Zhang, Hongli Liu, Ruitian Yi, Taotao Yan, Yingli He, Yingren Zhao, Jinfeng Liu

**Affiliations:** Department of Infectious Diseases, First Affiliated Hospital of Medical College, Xi’an Jiaotong University, Xi’an, 710061 Shaanxi Province China; Department of Gastroenterology, Xi’an Central Hospital, Xi’an, 710003 Shaanxi Province China; Xi’an Eighth Hospital Affiliated to Xi’an Jiaotong University Health Science Center, Xi’an, 710061 Shaanxi Province China

**Keywords:** HBV, HCC, HBV whole-X protein, HBx

## Abstract

**Background:**

The role of HBV X protein (HBx) in the development of hepatocellular carcinoma (HCC) has been well studied. However, little is known about the molecular functions of HBV whole-X protein (HBwx), a protein fused with HBx and upstream 56 amino acid, in HCC. In current study, the molecular functions of HBwx in HCC pathogenesis has been investigated, as well as comparison between HBwx and HBx.

**Methods:**

Expression of HBwx and HBx in 50 HCC tissues was examined by immunohistochemistry. Their tumor-forming abilities were evaluated by an animal model and colony formation assay. Migration and invasion were detected by transwell assay and subcellular localization was tracked by GFP fluorescence. Cell proliferation, cell cycle and apoptosis were detected by CCK8 and FCM. Protein level was determined by Western blotting.

**Results:**

HBwx was present in 72 % (36/50) of the liver tumor tissues and mainly expressed in the nucleus and deposited in the cytoplasm surrounding karyotheca. HBwx showed a promoting effect on tumorigenesis and growth in vivo and in vitro as well as cell migration and invasion, whilst such effect is compromised compared with that of HBx. Further analysis demonstrated differences in cell proliferation, cell cycle and cell apoptosis between cells expressing HBwx and those expressing HBx. Additionally, it was confirmed that RKIP-p-ERK pathway was involved in HBwx-related tumor formation.

**Conclusion:**

HBwx, with the extra 56 amino acids, is closely related with hepatocarcinogenesis, while displays different biological functions from HBx.

## Background

Chronic Hepatitis B virus (HBV) infection is a major risk factor of HCC and is reported to be associated with more than half of HCC cases worldwide [1, 2]. The HBx, coded by the HBV X gene, is believed to play a vital role in the pathogenesis of HCC by influencing cell cycle, proliferation, and apoptosis at the levels of cell signaling and transcription [3, 4]. Moreover, HBx also functions as a broad-acting transcriptional activator to promote the expression of a variety of cellular and viral genes including proto-oncogenes [5, 6].

Intriguingly, an additional open reading frame (ORF) with 56 nucleotide triplets upstream of the starting code of the X gene has been identified [7–10]. Our previous studies have displayed the novel ORF has transcriptional activity [11] and can be translated with the X gene in frame to form the HBV whole-X gene (wX) [12]. The HBV whole-X gene, which consists of the novel ORF and X gene and runs as long as 630 bp, codes the HBV whole-X protein composed of 210 amino acids. Studies have shown that the whole-X gene is relatively well conserved in numerous HBV genomes except for the start codon A nt 1205C/T or C/A nt 1330 T replacement mutation resulting in disability of coding whole-X gene. The whole-X gene is identified mainly in HCC patients in Asia or of Asian origin and is presented specifically in HBV C/adr genotype [13]. According to our knowledge, HBV C/adr genotype is more closely associated with HCC than any other genotypes. Loncarevic et al. reported that all the five HBV DNA clones derived from HCC patients had an intact whole-X ORF. Yang et al. demonstrated the interaction between HBwx and hepatoproteins by using a yeast two-hybrid assay, suggesting it may play a role in the development of carcinoma via modulating signal transduction of protein–protein binding in the liver cells [14]. These results implicate that HBwx, with extra 56 amino acids longer than HBx, may be related to HCC pathogenesis.

Whereas, the specific function of HBwx in HCC need further investigation. In this study, we examined the expression of HBwx in the liver tissues from patients with HBV-related HCC and investigated its influences on cell growth, cycle, apoptosis and oncological characters in comparison with HBx. We intended to reveal the functional differences between HBwx and HBx and to provide new insight into the possible role of HBwx in HCC pathogenesis.

## Methods

### Liver tissue specimens

Liver tumor specimens were obtained from 50 patients with HBV-related HCC, and the control liver tissues specimens were from 10 non-HCC patients with HBV infection after hepatectomy. The clinicopathological data of HCC specimens are presented in Table 1. The tissues were collected at the First Affiliated Hospital of Medical College, Xi’an Jiaotong University (Xi’an, China) from January 2002 to June 2009. The study protocols were approved by the Ethical Review Committee of the First Affiliated Hospital of Medical College, Xi’an Jiaotong University, and informed consent was signed by all participants. The methods were carried out in accordance with the approved guidelines.

**Table 1:** Correlation between HBwx expression and clinicopathologic features in 50 HCCs

Parameters	Number	HBwx Expression, *n* (%)	*P* value
Age (y)			0.796
≥55	13	9 (69 %)	
<55	37	27 (73 %)	
Gender			0.756
Male	44	32 (73 %)	
Female	6	4 (67 %)	
Histological grade			0.095
I + II	35	25 (71 %)	
III	15	7 (47 %)	
Tumor diameter (cm)			0.063
<5	22	18 (82 %)	
≥5	28	16 (57 %)	
AFP (ng/mL)			0.658
<400	29	19 (66 %)	
≥400	21	15 (71 %)	

### Preparation of HBwx polyclonal antibodies

Rabbit anti-HBwx antibodies were produced by commercial Company (Beijing Biosynthesis Biotech, Beijing, China). Peptides (12–14 amino acids) were designed specifically for the conserved regions of HBV *pre-X* (HBV DNA nt1207-nt1374) by protein sequencing and epitope analysis for antibody production. The peptides of two designed sequences (1^#^HAWNLCGSSADP, 2^#^YCGTPSSLFCSQPV) were synthesized and conjugated with KLH protein as the antigen. Immunized rabbit antiserums were collected and purified with antigen specific affinity purification, and then titered by Enzyme Linked Immunosorbent Assay (ELISA).

### Immunohistochemical staining (IHC)

Paraffin-embedded liver tissues were cut into 5 μm sections and placed on polylysine-coated glass slides. Antigen retrieval was achieved by pressure cooking for 2 min in citrate buffer (pH6.0). A rabbit anti-human HBwx polyclonal antibody at 1:1280 dilution and a mouse anti-HBx monoclonal antibody (ab235) (Abcam, Cambridge, MA) at 1:500 dilution were used as primary antibodies. Peroxidase-Conjugated AffiniPure Goat Anti-Rabbit IgG (ZB-5301) and Anti-Mouse IgG (ZB-5305) (Zhongshan Goldenbridge Biotech, Beijing, China) were used as the secondary antibodies. The substrate 3, 3′-diaminobenzidine tetrahydrochloride (DAB) was followed by counterstaining with hematoxylin. The negative staining control was performed with cold phosphate buffer solution (PBS) instead of the primary antibody. Immunostaining intensity of HBwx was divided into strong positive (++), scattered positive (+), seldom (±) and negative (−) according to the distribution of positive staining cells in the tissues by 2 independent observers.

### Plasmids

The full-length HBV *whole-X, X* genes were cloned from the plasma of the patients with chronic HBV infection, and subcloned into pcDNA3.1(−), pCMV-Tag2A and pEGFP-C1 vectors respectively. Recombinant plasmids pCMV-Tag2A-wX, pEGFP-C1-wX, pCMV-Tag2A-X and pEGFP-C1-X were further confirmed by DNA sequencing.

### Cell culture and transfection

Hepatoma cell lines SK-Hep-1 and SMMC-7721 cell lines were grown in Dulbecco’s modified Eagle’s medium (DMEM) (Gibco, Carlsbad, USA) supplemented with 10 % fetal bovine serum (FBS) (Gibco). HL-7702, a normal liver cell line (Shanghai Institute of Biochemistry & Cell Biology, Shanghai, China), was cultured in the RPMI-1640 medium supplemented 10 % FBS. Transfection was performed by using Lipofectamine 2000 (Invitrogen, Carlsbad, USA) according to manufacturer’s instruction. Cell lines stably overexpressing HBwx or HBx were established in SK-Hep-1cells by G418 (800/400 μg/ml) selection.

### Tumor formation in nude mice

18 Balb/c male nude-mice, 4–6 weeks old, were randomly divided into three groups, and then subcutaneously inoculated with 2 × 10^6^ transformed SK-Hep-1 cells containing pCMV-Tag2A-wX, pCMV-Tag2A-X and pCMV-Tag2A as a control. General state and tumor formation of mice were observed and recorded. The study protocol was approved by the Animal Research Committee of the Medical College of Xi’an Jiaotong University.

### Colony formation assay

The stably transfected SK-Hep-1 cells with overexpressing HBwx or HBx were seeded in 60 mm plates at a density of 500, 1000 or 2000 cells per plate. After 10 days incubation with DMEM medium containing 10 % FBS, the cells were fixed with 4 % paraformaldehyde for 30 min, stained with 0.1 % crystal violet for 20 min, and photographed. Three fixed-size areas were randomly chosen to count the colonies and the averages of colonies were calculated for different cell densities and cell lines.

### Cell migration and invasion assay

Cell migration and invasion assay for each overexpressing transformed cell line was performed by using 24-well Millicell (Millpore, Billerica, USA) coated without or with Matrigel (BD Biosciences, New Jersey, USA). 200 μl of 1 × 10^5^/ml cells were transferred onto the transwell chambers and cultured for 24 h allowing the cells to move through the extracellular matrix to the lower chamber. The cells on the underside of the inserts were fixed with 4 % paraformaldehyde for 30 min and stained with 0.1 % crystal violet. Each experiment was repeated at least three times independently. Five randomly selected fields on the fixed transwell chambers were counted with three repeats and photographed. Stained membranes were also discolored in 33 % HAc and absorbance of the elution solutions were measured in 96-well plates with a microplate reader (STAT FAX 2100, USA).

### Intracellular localization of HBwx

After being transiently transfected with Green Fluorescent Protein (GFP)-labeled recombinant pEGFP-C1-wX, pEGFP-C1-X and control plasmids, HL7702 cells were observed by fluorescence microscopy (microscope model Nikon Ti-s DS-Ril, Tokyo, Japan) at 48 h after transfection. SMMC-7721 with pCMV-Tag2A-wX, pCMV-Tag2A-X were fixed with 4 % paraformaldehyde for 10 min, permeabilized with 0.3 % Trition X-100 for 10 min, then incubated with anti-FLAG® M2 primary antibodies (F3165) (Sigma, St. Louis, USA) and FITC/TRITC fluorochrome-labeled secondary antibodies (ZF-0312/ZF-0313) (Zhongshan Goldenbridge Biotech). Cell nuclei were stained with 1 μg/ml DAPI for 3 min and then observed by fluorescence microscopy.

### Cell proliferation

The effects of HBwx and HBx on cell proliferation were evaluated by the Cell Counting Kit-8 assay (CCK-8) (Dojindo Laboratories, Kumamoto, Japan). 100 μl/well (5 × 10^3^) cell suspensions were inoculated in 96-well, incubated at 37 °C with 5 % CO_2_ for 24 h, 48 h, 72 h and 96 h. Then, the absorbance was measured at 450 nm using a microplate reader and cell proliferation rate relative to the control was calculated.

### Cell cycle and apoptosis

Cell cycle and apoptosis were observed using SK-Hep-1 cell lines stably transfected with pCMV-Tag2A-wX, pCMV-Tag2A-X and control plasmids by flow cytometry analysis (FCM). Cells were harvested (1-2 × 10^6^cells/ml) in logarithmic growth phase, washed with PBS and fixed in 70 % ethanol at 4 °C for at least 1 h. Then, the cells were washed in cold PBS, stained with 50 mg/ml propidium iodide (PI) in the darkness for 30 min and resuspended in PBS at 4 °C before being analyzed by CyFlow® SL (PARTEC Company, Germany). Annexin V-FITC/PI Apoptosis Detection kit (KeyGEN, Nanjing, China) was used to detect cell apoptosis rate induced by 0.1–2.0 μg/ml Adriamycin (ADM) and 0.5–2.5 μg/ml Lipopolysaccharides (LPS) at different concentrations and times (3 h, 6 h, 12 h and 24 h). FCM data were analyzed by using Muticycle AV and FloMax software supplied by the instrument.

### Western blot

Stably transfected cells were collected, and cell proteins were extracted by using whole cell extraction buffer (WCEB). Equal amounts of protein were separated in sodium dodecyl sulfate polyacrylamide gel electropheresis (SDS-PAGE) gel and transferred to Polyvinylidene Fluoride (PVDF) membranes (Millipore, Germany), then immunodetection was performed using monoclonal anti-Flag® M2 (F3165, Sigma), monoclonal anti-RKIP/PBP (ab76582, Abcam), polyclonal anti-p44/42 MAPK (Erk1/2) (#9102) and anti-Phospho-p44/42 MAPK (Erk1/2) (#9101) (Cell Signaling, Beverly, USA) antibodies.

### Statistical analysis

All statistical analyses were performed by using SPSS 13.0 software (SPSS Inc, Chicago, USA). Descriptive data were assessed using the Pearson’s chi-square test. The data from the experiments were shown as the mean ± SD. Analysis of Variance (ANOVA) was applied to determine the comparability of groups, *P* < 0.05 being considered statistical significance.

## Results

### Distinct expressions of HBwx and HBx in HBV-related HCC

**Fig. 1:**
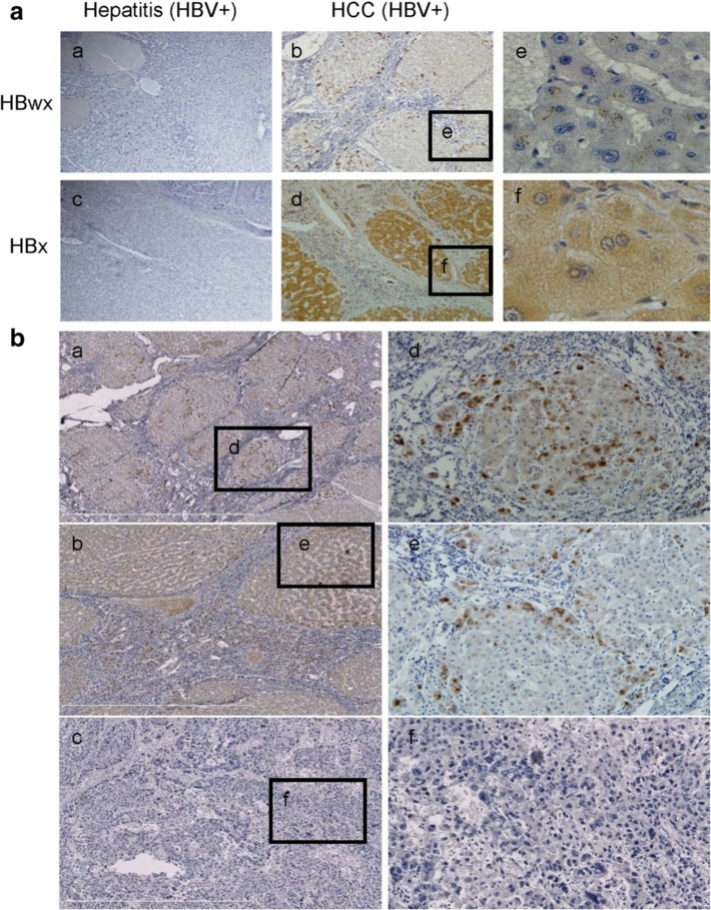
Subcellular distribution of HBwx and HBx and characteristics of HBwx distribution in HBV-associated HCC specimens detected by immunohistochemistry. **a** HBwx immunoreactivity was mainly observed in the nucleus and in the cytoplasm surrounding the nuclear membrane (*e*). Immunohistochemical staining of HBx revealed diffuse positivity in the hepatocytes, especially in cytoplasm (*f*) (*a*-*d*, original magnification, ×100) (*e*-*f*, original magnification, ×400). **b** More intense and widespread HBwx immunoreactivity was observed in the small tumor masses (*a*, *d*) or the peripheral parts of the large tumor masses near the portal area (*b*, *e*); No HBwx immunoreactivity was observed in tumor cells of poorly differentiated hepatocellular carcinoma (*c*, *f*) (*a*-*c*, original magnification, ×100) (*d*-*f*, original magnification, ×200)

The pathological role of HBwx in the development of HCC is largely unknown. To determine the involvement of HBwx in the pathogenesis of HCC, the expression of HBwx and HBx in the liver tissues from patients with the HBV-related HCC was examined by IHC. The results shown that HBwx was positively stained in 36 out of 50 liver tumor tissues (72 %) and staining grades of HBwx were 8 % +++, 24 % ++, 40 % + and 28 % -, whereas HBx was detected in all of these tissues (+++) (50/50). Next we further analyzed whether HBwx expression was correlated with clinicopathological characteristics. Our findings showed that no significant correlation between HBwx expression and clinicopathological features (Table 1). Interestingly, HBwx was distributed mainly in the nucleus of hepatocytes and around the nuclear membrane, whereas HBx was found to be almost evenly spreading in the cytoplasm of hepatocytes (Fig. 1a). Furthermore, the level of HBwx expression was significantly higher in small tumor masses of highly differentiated cells or peripheral parts of the large tumor masses near the portal area. HBwx was not detected in liver tumor of poorly differentiated cells and higher atypia (Fig. 1b). The data implicate that HBwx expression in liver tumor tissues with HBV infection is different from that of HBx and HBwx may be correlated with the progression and malignant degree of HBV-related HCC.

### Effects of HBwx on tumor formation of transformed cells in vivo and vitro

**Fig. 2:**
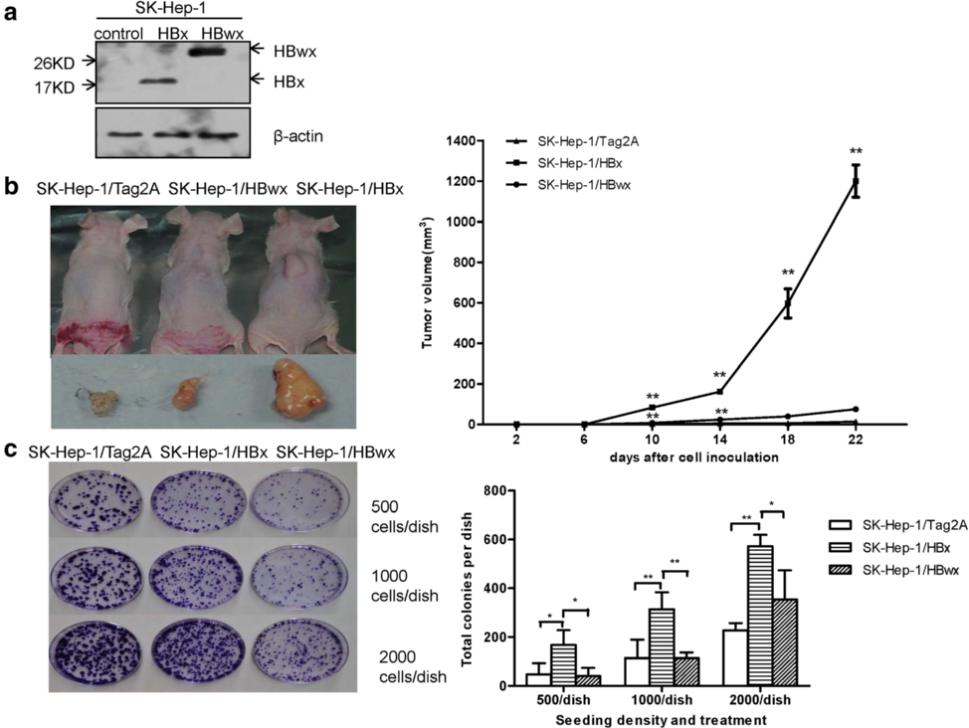
Effects of HBwx on tumor-forming abilities in vitro and vivo. **a** Stable expressions of HBwx and HBx were detected by Western blot in SK-Hep-1 cell lines. **b** Tumor formation in nude mice. SK-Hep-1-HBx, SK-Hep-1-HBwx and control cells were subcutaneously injected into BALB/c nude mice, and tumor growth was observed and recorded. Representative photographs of the gross neoplasms in nude mice after 22 days. Graphical presentation of the changes of tumor volume in mice after cell inoculation. Data were presented as means ± SD of six samples. Compared with the controls, HBwx significantly promoted tumor growth in nude mice (**P < 0.01), though its effect was less obvious than that of HBx. **c** Colony-formation assay was performed for the analysis of tumor-forming abilities in vitro. Colonies formed in the plates were photographed. Quantitative analysis of the total colonies in different seeding density and treatments. Data shown as mean ± SD of each group from three independent experiments. Compared with the control, overexpressed HBwx cells showed increased colony formation (**P < 0.01, *P < 0.05), though the increase was not as significant as that of HBx cells

In order to determine the different biological roles of HBwx and HBx, both proteins were overexpressed in SK-Hep-1 cells and stable cell lines were established (Fig. 2a).

Tumor formation in nude mice is often predictive of tumorigenicity in vivo. Therefore, we performed tumor formation in mice to determine the oncogenic effect of HBwx.

In this experiment, the cells with HBwx, HBx or an empty vector were injected subcutaneously into the front back of the nude mice. Tumor growth was monitored every four days with a caliper. These cell types showed a dramatic difference in tumor growth (Fig. 2b). When compared with HBx tumors, the formation of HBwx and vector control tumors was about 10 days delayed and the tumors grew markedly more slowly (SE = 0.86, *P* = 0.000). Gross examination of tumors from the HBx cells showed that they were firm, fleshy, rounded or lobulated grey-white masses, with an average volume of 1200 mm^3^ after 22 days of growth. Although the tumors formed from HBwx and the empty vector cells were much smaller, the SK-Hep-1/HBwx cells developed palpable tumors faster compared with SK-Hep-1/Tag2A cells. Significant difference was observed in tumor volumes between SK-Hep-1/HBwx and SK-Hep-1/Tag2A cells on the 10th (SE = 0.86, *P* = 0.001) and 14th day (SE = 4.92, *P* = 0.000). The average volume of HBwx and empty vector tumors was respectively 75.6 mm^3^ and 14 mm^3^. These results suggest that compared with HBx, HBwx may also contribute to the HCC pathogenesis, but to a lesser degree.

The above observation was further confirmed by the result in vitro. The same number of SK-Hep-1 cells overexpressing HBwx or HBx was seeded at different densities and the colonies were counted under a microscope. As shown in Fig. 2c, the number of colonies generated from cells with HBwx was significantly smaller than that from the cells with HBx, although the numbers of colonies from both cell types were larger compared with the control. The colony size from the cells with the empty vector was larger than that from the cells with HBwx or HBx. Quantitative analysis revealed that the number of colonies from HBx-overexpressing cells seeded in the three densities was respectively 4.2 (SE = 39.63, *P* = 0.019), 2.8 (SE = 49.89, *P* = 0.007), and 1.6 times (SE = 62.54, *P* = 0.013) that from HBwx-overexpressing cells. The results indicate that HBwx can promote colony formation in vitro, but not as significantly as HBx, suggesting that compared with HBx, HBwx may be a weaker tumorigenicity-promoting factor.

### Effects of HBwx on tumor character of transformed cells

**Fig. 3:**
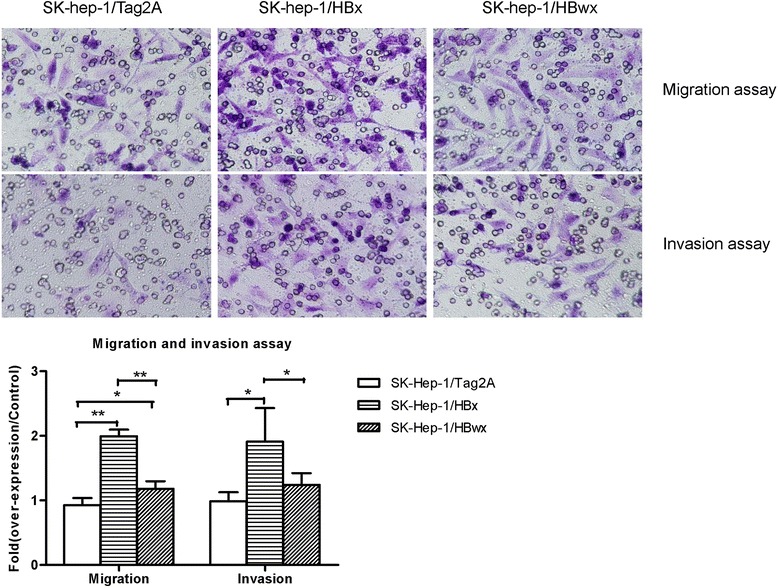
Cell migration and invasion assays: The experiments were performed in transwell chambers with non-Matrigel or Matrigel coated membranes by using stable transfected SK-Hep-1 cell lines with pCMV-Tag2A-wX, pCMV-Tag2A-X and control vectors. Cells were stained with crystal violet and photographed (magnification, ×200). Data shown as mean ± SD of each group from three independent experiments. Compared with the control cells, HBx cells showed significantly enhanced migration and invasion capacity (**P < 0.01, *P < 0.05), HBwx also showed a promoting effect on migration and invasion. However, the migratory and invasive ability of HBwx cells was significantly reduced compared with HBx cells (**P < 0.01, *P < 0.05)

Migration and invasion are the basic character of malignant tumor cells. To determine the different effects of HBwx and HBx on migration and invasion, we performed transwell assays by using cells overexpressing HBx or HBwx. Interestingly, cells overexpressing HBx migrated (SE = 0.09, *P* = 0.000) and invaded (SE = 0.27, *P* = 0.047) significantly faster than cells overexpressing HBwx, though compared with the control, HBwx also showed a promoting effect on migration (SE = 0.09, *P* = 0.030) and invasion (Fig. 3). Under the same condition, the number of migrated and invaded cells in cells overexpressing HBx was respectively 2.1 and 1.9 times that in the control, whereas the number of both migrated and invaded cells in cells overexpressing HBwx was 1.3 times that of the control. These results indicate that though HBwx can stimulate migration and invasion, its performance is significantly weaker than that of HBx.

### Different subcellular localization of HBwx and HBx

**Fig. 4:**
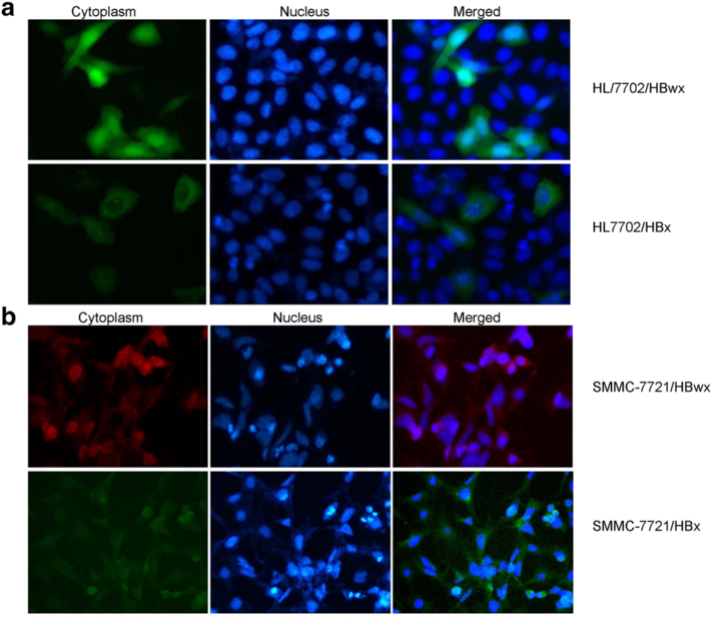
Subcellular localization of HBwx and HBx in transfected cells. **a** EGFP fused HBwx or HBx protein in HL7702 cells was observed respectively by fluorescent microscopy; **b** SMMC-7721 cells transiently transfected with pCMV-Tag2A-wX or pCMV-Tag2A-X and control were observed by cell immunofluorescence staining. Both results showed that HBwx was mainly expressed in the nucleus, whereas HBx in the cytoplasm

Based on the observations above, we presumed that HBwx and HBx might have different biological functions in the pathogenesis of HBV-related HCC. To determine the possible difference, we investigated intracellular localization of both proteins in different cell types. HL7702 cells were transiently transfected with GFP fused HBwx or HBx plasmids. As shown in Fig. 4a, HBwx was expressed chiefly in the nucleus whereas HBx was mainly in the cytoplasm, which was consistent with the observations in the tissue by IHC. The intracellular localization of HBwx was further confirmed by using different cell types by immunofluorescence staining and similar results were obtained (Fig. 4b). Overtly, the difference between HBwx and HBx in cellular localization may suggest their different pathogenic roles in formation of HBV-related HCC.

### Influences of HBwx overexpression on cellular biologic behaviors

**Fig. 5:**
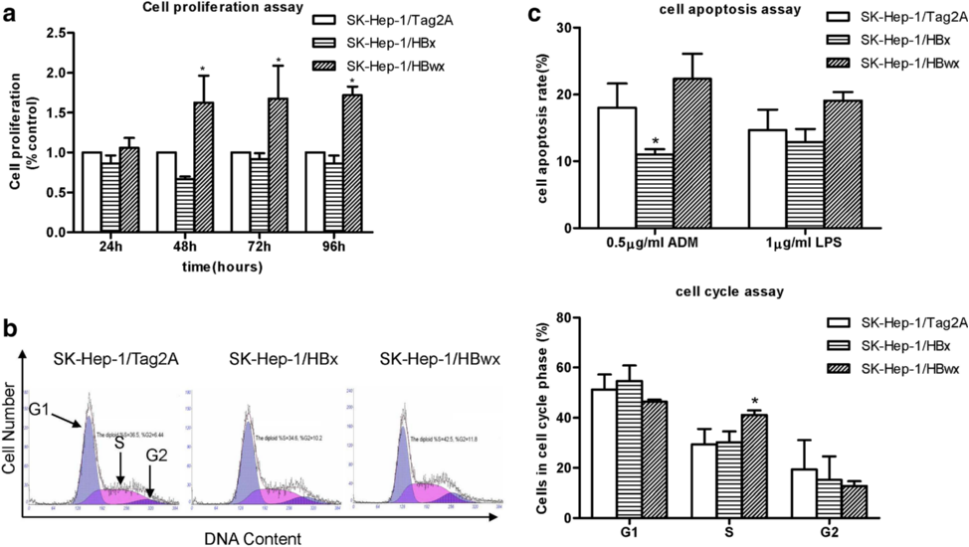
Effects of HBwx on cell biologic behaviors. **a** HBwx promoted cell proliferation (*P < 0.05). Cell proliferation was determined in transformed SK-Hep-1 cell lines by CCK-8 assay. Data were presented as mean ± SD (% relative to control) of each time point from three independent experiments. **b** Cell-cycle distribution was examined by PI staining and flow cytometry assay. Results were visualized as a representative experiment or means ± SD of three experiments. HBwx expression stimulated an increase in the proportion of cells at the S phase (41.2 %) compared with HBx overexpressing cells (30.2 %) and control cells (29.4 %) (*P < 0.05). **c** HBwx was associated with stronger apoptotic effect even with weak apoptosis stimulation. Apoptosis rates were determined by annexin V and PI staining using FACS analysis induced by ADM and LPS at different concentrations and time points. The transformed cell lines showed differential cell apoptosis rates. With 0.5 μg/ml ADM and 1.0 μg/ml LPS, the apoptosis rate of overexpressed HBwx cells was higher than that of HBx cells at 6 h

The effect of HBwx and HBx on cell proliferation was analyzed by using CCK-8 assay. As shown in Fig. 5a, HBwx significantly promoted cell proliferation (*P* < 0.05).

In contrast, HBx slightly inhibited cell proliferation without significant difference between SK-Hep-1/Tag2A and the SK-Hep-1/HBx cells. We also examined whether HBwx and HBx impact on cell cycle. Apparently, the number of cells at the S phase in the HBwx overexpressing cells were significantly higher (41.2 %) than that in the HBx overexpressing cells (30.2 %) or in the control cells (29.4 %) (SE = 3.68, *P* = 0.019). In contrast, the cell population at the G2/M phase was similar among the three cell types. There was no statistical significance between the cell-cycle distribution of SK-Hep-1/Tag2A cells and SK-Hep-1/HBx cells (Fig. 5b). Surprisingly, HBwx overexpressing cells were more sensitive to ADM and LPS induced apoptosis. The cells were treated with 0.5 μg/ml of ADM and 1 μg/ml of LPS for 6 h. The percentile of apoptotic cells after treatment with ADM and LPS was respectively 22.37 and 19.1 % in the HBwx overexpressing cells, 11 % (SE = 2.49, *P* = 0.031) and 12.9 % in the HBx, and 18 and 14.65 % in the empty vector control (Fig. 5c).

### Tumor associated signal molecules activated by HBwx

To explore the role of HBwx in cell functions, we performed proteomic analysis to examine the differential expression of proteins in cells overexpressing HBwx.

As we described in [15], 18 proteins were found to be obviously up or down regulated in the cells with HBwx, compared with the cells with an empty vector. These proteins are involved in transcription regulation, receptor binding, metabolism, apoptosis and signal transduction. Among them, the Raf-1 kinase inhibitory protein (RKIP) is well known to be closely associated with HCC pathogenesis. The expression of RKIP was significantly decreased in both HBwx and HBx overexpressing cells compared with that in the cells with an empty vector control.

**Fig. 6:**
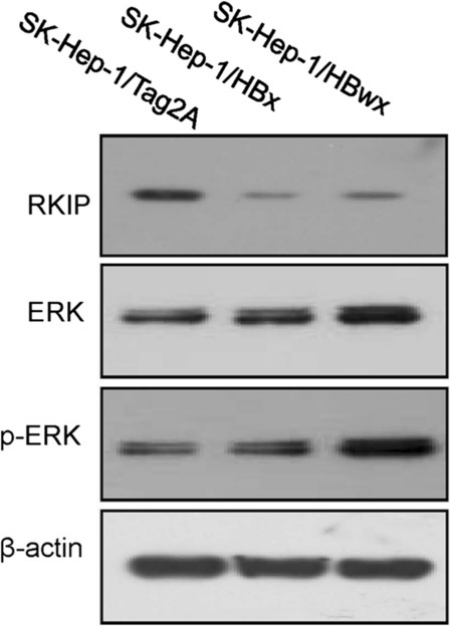
HBwx promoted activation of ERK by inhibiting the RKIP expression. Expressions of RKIP, ERK, and p-ERK in stable transfected cell lines with HBwx and HBx were detected by Western blot. In both HBwx and HBx cells, RKIP expression was donwregulated, p-ERK expression was upregulated

It has been well known that RKIP inhibits the Ras-Raf-1-MEK1/2-ERK1/2 pathway. To determine if the down regulation of RKIP impacts the Mitogen-Activated Protein Kinase (MAPK) pathway, we examined the activation of Extracellular Signal Regulated Kinase (ERK). As expected, the level of phopho-ERK (p-ERK) was notably higher in the cells with HBwx or HBx (Fig. 6), suggesting that HBwx contributes to tumor formation via mediating the MAPK pathway.

## Discussion

HBV is the smallest human hepatotropic DNA virus, which mainly infects host hepatocytes and causes a spectrum of pathological processes from acute hepatitis, chronic hepatitis to serious end-stage liver diseases such as hepatic cirrhosis and primary hepatocellular carcinoma [16]. Studies of epidemiology and natural history have provided a line of evidence that approximately 25 % chronic HBV-infected individuals will develop HCC [2, 17, 18]. Although the pathogenesis of HBV-related HCC is still elusive, HBx has been well documented as a risk factor and strongly implicated in hepatocarcinogenesis. HBx contributes to HBV replication and interacts with host factors to be involved in gene expression, cell proliferation, cell cycle progress, apoptosis, DNA repair and genetic stability [6]. Most importantly, HBx functions as a broad-acting transcriptional transactivator, promoting the expression of a variety of proto-oncogenes including c-myc, c-fos, and c-jun etc. [19].

Since the identification of the HBwx gene [7–12], previous studies including ours, have shown that HBwx may be associated with HCC [8, 10]; whereas, its physiological role is largely unknown. In this study, HBwx was expressed in about 72 % liver tumor tissues of patients with HBV-related HCC. Interestingly, small tumor masses contain more HBwx positive cells. In contrast, a significantly smaller number of HBwx positive cells were found in large tumor masses and in tumor with higher atypia. Additionally, HBwx displayed a promoting effect on tumorigenesis and growth in vivo and in vitro as well as cell migration and invasion. Consistent with our published data [15, 20], those results provided substantial evidence for the involvement of HBwx in hepatocarcinogenesis.

However, the subcellular location of HBwx and HBx in the liver cells is distinct from IHC with tumor tissue and subcellular localization analysis. It is generally accepted that the molecular function of a protein is highly dependent on its subcellular localization. By proteomics and bioinformatics analyses, our previous studies have proved that HBwx functions in carcinogenesis in a way that is different from that of HBx [20]. To discover the differential biological role of HBwx and HBx, the influence of HBwx on cellular biologic behaviors had been evaluated. In comparison with HBx,

HBwx displayed a higher cell proliferation rate, higher percentage of cells at the S phase, more sensitive to cell apoptosis stimulants. The observation implicates that HBwx may be a weaker tumorigenicity-promoting factor compared with HBx. However, there is no significant correlation between HBwx expression and clinicopathological characteristics of HCC. The differences between HBwx and HBx in hepatocarcinogenesis need thorough mechanism research and a large number of clinical samples to clarify. The present results provide significant information for understanding the mechanism of hepatocarcinogenesis and demonstrate clinical implications that may influence diagnostic decisions and treatment strategies in individual patient.

The differential expression of proteins in the cells with HBwx was investigated by proteomic analyses [15]. Our data suggest that the HBV whole-X may promote the development of liver cancer through downregulating the expression of RKIP, an inhibitor of the Ras-Raf-1-MEK1/2-ERK1/2 pathway. RKIP expression has been reported to be lost or reduced in prostate cancer, breast cancer, liver cancer and other malignant tumors because its role in inhibiting the RAS/RAF/MEK/ERK signaling pathway [21], which is activated in over 90 % of HCCs. A substantial evidence has manifested the activation of HBx in hepatocarcinogenesis [22, 23]. In our observation, the expression of RKIP was inhibited both in cells expressing HBwx and HBx, with increasing p-ERK, which implicates the involvement of RKIP-p-ERK pathway in HBwx carcinogenesis. Nevertheless, the explicit biological role and the specific molecular mechanism of HBwx, such as the effects of proliferation, metastasis and angiogenesis induced by HBwx remain to be studied, further investigation on the detail biological function is carrying out.

## Conclusions

This study expand our knowledge about the biological function of HBwx in HBV-related HCC. Whereas, the extra 56 amino acids endowed HBwx with distinct pathogenesis from HBx, and the specific mechanism of HBwx need further investigation.

## Abbreviations

HBV, Hepatitis B virus; HBwx, HBV whole-X protein; HBx, Hepatitis B virus X protein; HCC, hepatocellular carcinoma
